# *In Vitro* Culture Conditions and *OeARF* and *OeH3* Expressions Modulate Adventitious Root Formation from *Oleaster* (*Olea europaea* L. subsp. *europaea* var. *sylvestris*) Cuttings

**DOI:** 10.1155/2014/974086

**Published:** 2014-01-23

**Authors:** Adriana Chiappetta, Cinzia Gagliardi, Leonardo Bruno, Maria Beatrice Bitonti

**Affiliations:** Department DiBEST, University of Calabria, Ponte P. Bucci, Arcavacata di Rende, 87036 Cosenza, Italy

## Abstract

*Olea europaea* L. subsp. *europaea var*. *sylvestris*, also named *oleaster*, is the wild form of olive and it is used as rootstock and pollen donor for many cultivated varieties. An efficient procedure for *in vitro* propagation of oleaster was established in this study. A zeatin concentration of 2.5 mg/L was effective to induce an appreciable vegetative growth. Also high rooting efficiency was obtained by using a short IBA pulse, followed by two different IBA concentrations in the culture medium. With the aim to enlarge knowledge on the molecular aspects of adventitious rooting, we also evaluated the transcriptional modulation of an *ARFs* member and *HISTONE H3* genes, involved in auxin signaling and cell replication, respectively, during the root induction phase of cuttings. The obtained results suggest that the selected genes, as markers of the induction phase, could be very useful for setting up efficient culture conditions along the rooting process, thus increasing micropropagation efficiency.

## 1. Introduction

*Olea europaea* L. subsp. *europaea* var. *sylvestris* (Hoffm *et* Link), commonly named *oleaster*, is a widespread component of evergreen vegetation that extends along the coastal and subcoastal areas in the Mediterranean. Such vegetation, commonly named “*Macchia*” in the Italian language, consists predominantly of sclerophyllous shrub formations suitable to tolerate the arid conditions that mark the Mediterranean region. In this landscape, “*Macchia*” formation plays a major role in protecting soil from erosion and ensuring an idoneous hydrogeological structure as well as in reducing the outflow and nutrient losses [1, 2]. Moreover it is characterized by a high degree of biodiversity and it is rich in endemic species, many of which are source of valuable products such as honey, liqueurs, fruits, herbs, and numerous medicinal substances.

Since the last 50 years, “*Macchia*” formation is undergoing to erosion processes in the entire Mediterranean basin due to the worldwide increase of desertification threat and an unsustainable exploitation of land, which are causing a depletion of natural resources [3, 4]. Currently, “*Macchia*” vegetation appears highly fragmented and replaced, in most cases, by arid fields, eroded soils, and bare rock which strongly contribute to hydrogeological unsettlement of coasts. In this context, a recovery of the coastal landscape through an efficient and rapid restoration of autochthonous plant formations in the degraded areas represents an urgent and challenging task.

As previously mentioned, *oleaster*, which represents the wild form of olive, is largely prevalent in the Mediterranean vegetation due to a high photosynthetic efficiency and drought tolerance, related to its tap-root system that allows a deep exploration of the soil and an easy implant on both sandy soils and rocks [5, 6]. So far, *oleaster* is only used as rootstock and pollen donor for many cultivated varieties of olive, while due to the above mentioned features it could be widely applied for restoring eroded soils through vegetative propagation. Unexpectedly, despite its potential, wild olive aroused scarce interest among scientific community involved in research on *Olea* species, and mainly with respect to vegetative propagation capacity.

Vegetative propagation is extensively used in agriculture, horticulture, and forestry for multiplying selected plants from natural or inbreeded populations [7]. Among the different approaches, vegetative propagation by cuttings is one of the most common. Therefore, for any species, a lack of competence to form adventitious roots by cuttings is an obstacle for applying successfully vegetative propagation approach [8, 9].

Cutting rooting is a complex and critical process controlled by both endogenous and environmental factors such as phytohormones, wounding and light. The superroot *Arabidopsis* mutants accumulate indole-3-acetic acid (IAA) and develop numerous adventitious roots on the hypocotyl [10, 11]. *pin-formed1* rice mutants, defective in auxin polar transport, are affected in adventitious root emergence, thus confirming the importance of auxin concentration and distribution [12]. Adventitious root formation from stem cuttings of *Ginkgo biloba* and growth and survival of derived plantlets is enhanced by IBA treatment [13]. It is known that auxin can rapidly modulates the transcript levels of numerous genes through the activity of two related gene families such as *AUXIN RESPONSE FACTORs* (*ARFs*) and *AUXIN/INDOLE-3-ACETIC ACIDs* (*Aux/IAAs*) [14, 15]. In particular, ARFs bind to the auxin response elements (AuxREs) in the promoter region of early auxin response genes and activate or repress their transcription [15]. Therefore, the auxin may directly induce changes in gene expression.

Besides auxin concentration and distribution, also tissue sensitivity to this chemical compound, as well as explant physiological status and interaction with other factors, such as light, appear relevant for rooting process [16, 17]. De Klerk [18] showed that apple cuttings are sensitive to auxin after stem cell activation by starch, which occurs during the first 24 h and became committed to the formation of root primordia starting from 72 to 96 h of culture, through the interaction with light. Also in *Pinus sylvestris* light sources with different spectra enhance *in vitro* adventitious root formation [19].

In line with rooting being a critical step in vegetative propagation by cuttings, in our previous work an effective vegetative growth was set up for seedling-derived cuttings of *oleaster*, while rooting performance was not jet fully satisfactory. Therefore the present work was first of all addressed to improve vegetative propagation of *oleaster* cuttings, mainly with respect to the rooting phase.

In this context, it is worthy to recall that adventitious rooting is accomplished through different steps. The first step, named root induction phase, is the onset of cell proliferation that occurs via a serie of biochemical regulatory events, culminating in cell progression through cell cycle. Subsequently, it follows the root initiation and protrusion phases, corresponding to the first anatomical modifications and to root primordia emergence, respectively [17, 20]. So far, despite the large knowledge at the morphoanatomical level, molecular mechanisms that underlie adventitious root formation are not yet fully understood and mainly in woody plants [21–24]. Conceivably, the initiation and transition between the different phases of root development requires changes in the balance of expression of many genes, underlying the onset of cell division and the changes in cell differentiation pattern. Certainly, cell cycle progression involves the activation of genes encoding enzymes used in nucleotide metabolism and DNA synthesis, as well as of histone genes that produce proteins required for the packaging of newly replicated DNA. Accordingly, it has been reported that both in *Pinus contorta* and *Oryza sativa* the expression level of the S-phase-specific histone *H3* gene increased during the induction of adventitious root primordia [21, 25].

On these bases, aiming also to enlarge knowledge on the molecular aspects of adventitious rooting, we planned to evaluate the transcriptional modulation of an *ARFs* member and *HISTONE H3*, involved in auxin signaling and cell replication, respectively, during the root induction phase of *oleaster* cuttings cultured under different conditions.

## 2. Materials and Methods

### 2.1. Plant Material

Seeds (*n* = 500) of *Olea europaea* subsp. *europaea* var. *sylvestris* were collected from *sylvestris* plants growing in open field at the locality “*Pietra del Demanio*” in Cosenza, Italy, and vernalized for 3 weeks at 4°C [26]. After mechanical removal of endocarp, they were soaked for two days, sterilized with 70% alcohol for 1 min and then treated with sodium hypochlorite 5% (v/v) plus tween 20 0.1% (v/v) for 12 min. Seeds were rinsed five times with sterile distilled water, treated with the “Plant Preservative Mixture” (PPM) 1.5% (v/v) (Micropoli, Milan, Italy), supplemented with 50 mg/L of magnesium salts (chloride magnesium, sulphate magnesium, and nitrate magnesium). The PPM treatment was performed for 7 hours under continuous stirring. Finally, seeds were placed in culture on a substrate consisting of sterile water and bacto-agar 0.7% (w/v), pH 5.8. Seeds were kept at 24 ± 1°C under 16 h light per daily photoperiod. Irradiance intensity during the light period was 55 *μ*mol m^−2^ s^−1^ PAR obtained by a cool-light fluorescent lamps. To induce a rapid vegetative growth, after 30 days of culture, root-excised seedlings were transferred to olive medium (OR) [27], enriched with 30 g L^−1^ mannitol, 5 mg/L (23 *μ*M) *trans*-zeatin (Sigma, Milan, Italy), 0.8 g L^−1^ bacto-agar, and 0.1%. PPM [27, 28]. This medium was named ORZ_5_.

### 2.2. Micropropagation Experiments

After 45 days on the ORZ_5_ medium, cuttings with 4-5 mm in length were excised and used for micropropagation experiments. These cuttings will be referred to as Sc_s_ (Seedling derived cuttings). In particular, *n* = 200 single node cuttings without the apical bud were transferred on Murashige and Shoog (MS) and OR medium. Each medium was supplemented with 30 g L^−1^ mannitol, 2.5 mg/L (11.5 *μ*M), or 5 mg/L (23 *μ*M) *trans*-zeatin (Sigma, Milan, Italy), 0.8 g L^−1^ bacto-agar, and 0.1% PPM. These mediums were named MSZ_2.5/5_ and ORZ_2.5/5_. The pH was adjusted to 5.8 before autoclaving. Three independent replicates were performed, and subcultures were made after 45 days. Sc_s_ vegetative growth was monitored by using different parameter: *budding percentage* (= number of cuttings with open buds/total number of cuttings × 100), *shooting percentage* (= number of cuttings with lateral shoots/total number of cuttings × 100), and shoot *length*.

### 2.3. Rooting Experiments

To improve rooting capacity of Sc_s_ and above all to shorten the period for inducing adventitious root formation, after 60 days on the ORZ_5_ medium, *n* = 100 shoot apical meristem with three nodes below were excised and transferred to OR modified medium, supplemented with 30 g L^−1^ sucrose, 2.5 mg/L phytagel, PPM 0.1%, and pH 5.8. The medium used was enriched or not with IBA at different concentrations.

Root induction was performed by the followed scheme, according to Peixe et al. [29]:

- direct inoculation of the explant basal portion into the OR modified medium without IBA (*T*_1_);
- short pretreatment (20 s), into a 14.7 *μ*M IBA sterile solution followed by inoculation of the explants into the OR modified medium, supplemented with IBA 0.5 mg/L (2.4 *μ*M) (*T*_2_);
- short pretreatment (20 s), into a 14.7 *μ*M IBA sterile solution followed by inoculation of the explants into the OR modified medium, supplemented with IBA 1 mg/L (4.9 *μ*M) (*T*_3_).

With the aim to obscure the medium surface, a “darkening solution” was added to solidified OR modified medium. The “darkening solution” was composed of 4 g L^−1^ activated charcoal, 0.8 g L^−1^ bacto-agar, and PPM 0.1%.

Samples were harvested at 0 and 4 days. The last time represent the first stage of root development, corresponding to the root “induction” phase while the emergence of adventitious roots through the cutting epidermis was 6–8 days after treatment. The experiments were performed two times and, for each treatment, twenty-five samples were analyzed.

### 2.4. Histological Analyses

Adventitious root primordia were excised after 28 days of *in vitro* culture and root tips (*n* = 5) 1 cm long were fixed in 3% (w/v) paraformaldehyde and 0.5% (v/v) glutaraldehyde in PBS buffer (135 mM NaCl, 2.7 mM KCl, 1.5 mM KH_2_PO_4_, 8 mM K_2_HPO_4_, and pH 7.3) for 3 h at 4°C. After washing in the same buffer, samples were dehydrated and embedded in Technovitt 8100 resin. Semithin sections (4 *μ*m) were obtained using an ultracut microtome (Leica RM 2155) and stained with periodic acid-Schiff's reagent and 0.5% (w/v) Azur II. The adventitious root anatomy was compared with embryonic one.

### 2.5. RNA Isolation and sscDNA Synthesis

Total RNA was isolated from 100 mg of explant bases of Sc_s_, ~5-6 mm, induced to rooting as described above. RNA isolation was performed using the RNeasy Plant Mini kit (Qiagen, Hilden, Germany) as previously described: Bruno [30]. RNA was suspended in RNase-free water (50 *μ*L), treated with DNase I (100 *μ*L final volume) at 37°C for 50 min, reprecipitated and concentrated (40 *μ*L). The RNA was measured by the NanoDrop Spectrophotometer ND-1000, and quality was checked by electrophoresis evaluating the 28S rRNA and 18S rRNA ratios. Single-strand cDNA was synthesised from total RNA (3–5 *μ*g) by the SuperScript III Reverse Transcriptase and the oligo dT_(20)_ following the manufacturer's instructions (Invitrogen, Milan, Italy).

### 2.6. cDNA Library Generation

The genes investigated were present in a cDNA library generated from 50 to 100 *μ*g of total RNA extracted from leaves of* Olea europaea* subsp.* europaea* var. *sylvestris* plants, using the SMART system and cloning the sequence (around 1.2 kb) in the pSPORT1 vector. The sequencing analysis was performed from 5′ end. Generation and sequencing of the library was performed by Eurofins MWG GmbH cDNA Laboratory Fraunhoferstr (De) service. The GenBank accession number of *OesARF*, *OesH3*, and *OesH2b* cDNAs and genomic sequences are under submission.

### 2.7. Quantitative Real-Time PCR (qRT-PCR)

Gene expression analysis was performed by quantitative real-time PCR on a STEP ONE (Applied Biosystems) single colour thermocycler, with *Power SYBR Green PCR Master Mix 2X* (Applied Biosystem) (Cat. no. 4368702).

The oligonucleotide primer sets used for qRT-PCR analysis were designed using Primer3 (http://primer3.ut.ee/) according to the strategies set up by [31]. The primers used for qRT-PCR were *OesARF* FW 5′-TGAGACCCAAAAAGGACCAC-3′ and *OesARF* BW 5′-CTTCCCTCCACTTGGGTTCT-3′; *OesH3* FW 5′-CTACCATTCCAGCGTTTGGT-3′ and *OesH3* BW 5′-GACCCACAAGGTAAGCCT-3′. The olive histone *H2b* was used as a normalization control according to De Almeida et al. [24]. The *OesH2b* primer sequences were *OesH2b* FW 5′-CTCGGGAGATTCAGACTGCT-3′ and *OesH2b* BW 5′-TTCATCAATTCAGGAGCTGGT-3′. Amplification reactions were prepared in a final volume of 25 *μ*L by adding 12.5 *μ*L of the iTaq SYBR-Green Super Mix with ROX (Bio-Rad), 1 *μ*L (0.4 *μ*L M) of primers, and 2 *μ*L (25 ng) of cDNA.

All reactions were run in triplicate, in 48-well reaction plates, and negative controls were set. The cycling parameters were as follows: one cycle at 95°C for 10 min to activate the Taq enzyme, followed by 40 cycles of denaturation at 95°C for 15 s, and annealing-extension at 58°C for 30 s. To confirm the occurrence of an unique PCR product, the “melting curve” [32] was evaluated by an increase of 0.5°C every 10 s within a 60 to 95°C range and an unique “melting peak” in every reaction was observed. The quantitative qRT-PCR data were analyzed using STEP One Software 2.0 (Applied Biosystems) with the 2^−ΔΔCT^ method [33]. The means of *OesARF* and *OesH3* expression levels were calculated from three biological repeats, obtained from three independent experiments.

### 2.8. Statistical Analysis

The results are reported as the mean values obtained from two independent replicate. Significant differences among samples were determined by analysis of variance (ANOVA) followed by Turkey's Honestly Significant Difference (HSD) test, using the IBM SPSS statistics professional edition software 11.0 for Windows.

## 3. Results

### 3.1. Induction of Sc_s_ Vegetative Growth

**Figure 1:**
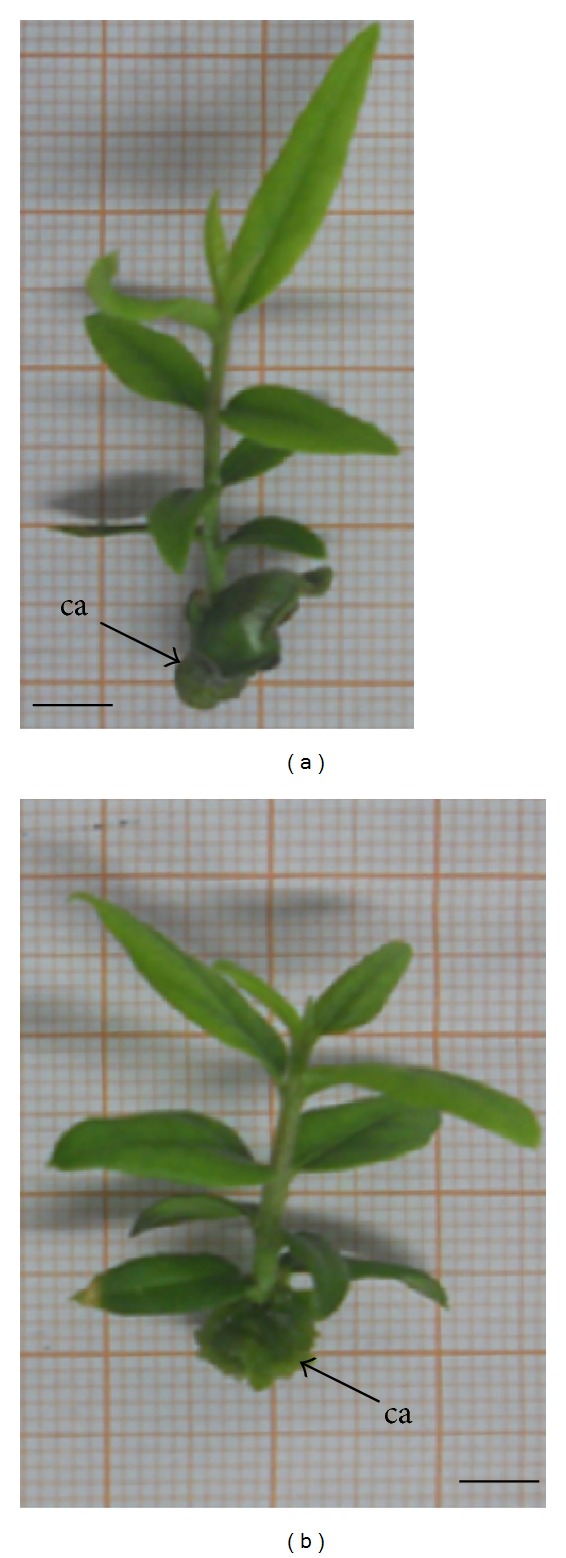
Vegetative growth of seedling-derived cuttings (Sc_s_) of *Olea europaea* L. subsp. *europaea* var. *sylvestris* after 45 days on ORZ_5_: olive medium + 5 mg/L (23 *μ*M) *trans*-zeatin (a) and (b) MSZ_5_: Murashige and Shoog medium 5 mg/L (23 *μ*M) *trans*-zeatin, respectively. ca: callus. (a), (b) = 0.8 mm.

Single node Sc_s_ were explanted on two different media enriched with either 2.5 mg/L (11.5 *μ*M) or 5 mg/L (23 *μ*M) *trans*-zeatin (Sigma, Milan, Italy) (i.e., MSZ_2.5/5_ and ORZ_2.5/5_). Appreciable percentages in budding and shooting processes were obtained whatever growth condition was applied (Table 1). However, on both growth media, budding and shooting percentages were higher at 2.5 than 5 mg/L zeatin concentration. In addition, abundant callus formation occurred at the basal end of cutting on medium enriched with the highest hormone concentration (Figure 1).

Irrespective of hormone concentration, the proportion of Sc_s_ forming budding was quite similar on MSZ and ORZ medium, while percentages observed in the shooting process were lightly but significantly higher on ORZ *versus* MSZ medium. However, different media did not influence the length of developed shoot (Table 1).

Irrespective of media used, *in vitro* cultures did not show any symptom of vitrification.

### 3.2. IBA Requirement for Adventitious Rooting

Actively growing Sc_s_ were then transferred to rooting medium. With the aim to obtain a high rooting efficiency and above all to shorten the period for inducing adventitious root formation, different protocols were applied, as described in Section 2.

Sc_s_ were maintained on rooting medium for 6 weeks and daily monitored in the first 12 days; thereafter observations were performed each week. Root protrusion was observed not before 6–8 days after placing in rooting medium. The reported results deal with 28 days of culture. In all the replicates, prolonged rooting phase did not give higher performance.

**Figure 2:**
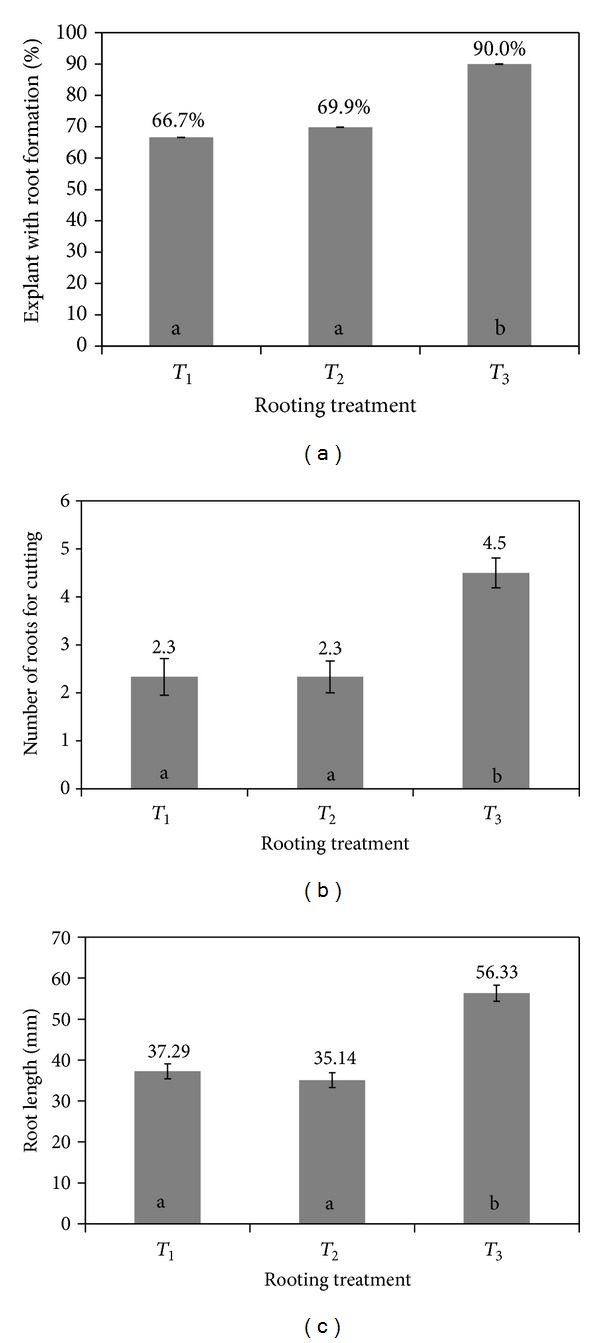
Adventitious rooting percentage (number of rooted cuttings/total cuttings in culture × 100) (a), number of roots for cutting (b), and root length (c) on Sc_s_ of *Olea europaea* subsp. *europaea* var. *sylvestris* grown on different medium for 28 days. *T*_1_: OR medium without IBA; *T*_2_: short pretreatment with 14.7 mM IBA sterile solution and inoculation into the OR medium supplemented with 0.5 mg/L IBA; *T*_3_: short pretreatment with 14.7 mM IBA sterile solution and inoculation into the OR medium supplemented with 1 mg/L IBA. The results represent the average value of two independent replicate. Means with common letters are not significantly different at *P* ≤ 0.05, according to Turkey's Honestly Significant Difference (HSD) test.

At this time, the proportion of Sc_s_ forming root was 66.7% when they were transferred on an auxin-free medium (*T*_1_). A quite similar result was obtained for Sc_s_ pretreated with IBA pulse and then transferred on a medium supplemented with the lower auxin concentration (*T*_2_). On the contrary, the proportion of Sc_s_ forming roots increased to 90% when IBA pulse was associated with the highest auxin concentration (*T*_3_) (Figure 2(a)).

A similar pattern was observed with respect to the number of roots per cutting (Figure 2(b)) and the root lenght (Figure 2(c)). Namely, the values of both these parameters did not differ at *T*_2_ condition compared to the auxin free medium (*T*_1_) and peaked at *T*_3_ condition (Figures 2(b) and 2(c)).

**Figure 3:**
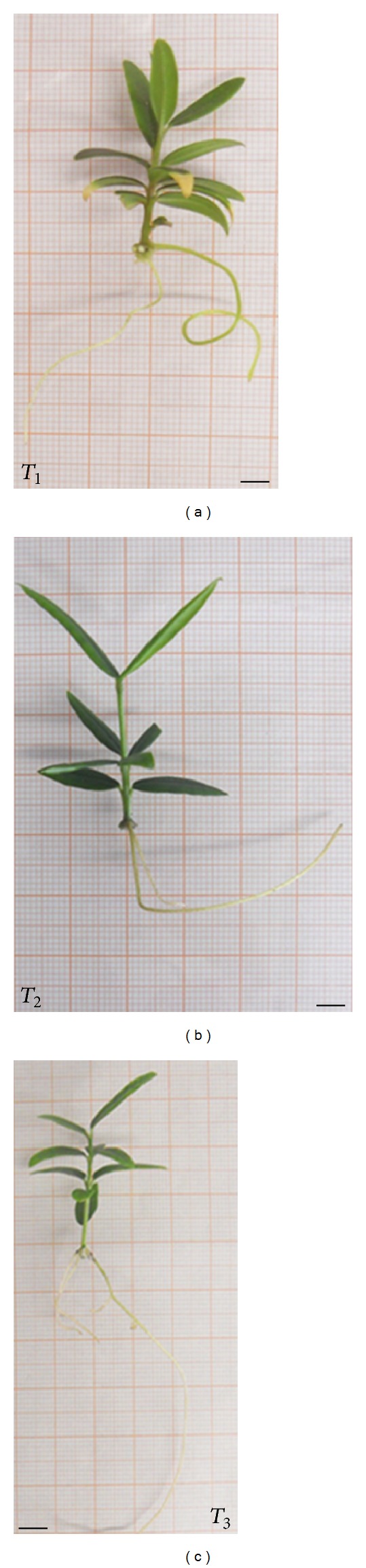
Effect of different IBA concentration on adventitious root morphology of *Olea europaea* subsp. *europaea* var. *sylvestris* grown on different medium. *T*_1_: OR medium without IBA; *T*_2_: short pretreatment with 14.7 mM IBA sterile solution and inoculation into the OR medium supplemented with 0.5 mg/L IBA; *T*_3_: short pretreatment with 14.7 mM IBA sterile solution and inoculation into the OR medium supplemented with 1 mg/L IBA. (a) = 1.9 mm; (b) = 1.3 mm; (c) = 1.9 mm.

The described differences in root number and length are clearly illustrated in Figure 3.

### 3.3. Histological Analyses of Adventitious Root

**Figure 4:**
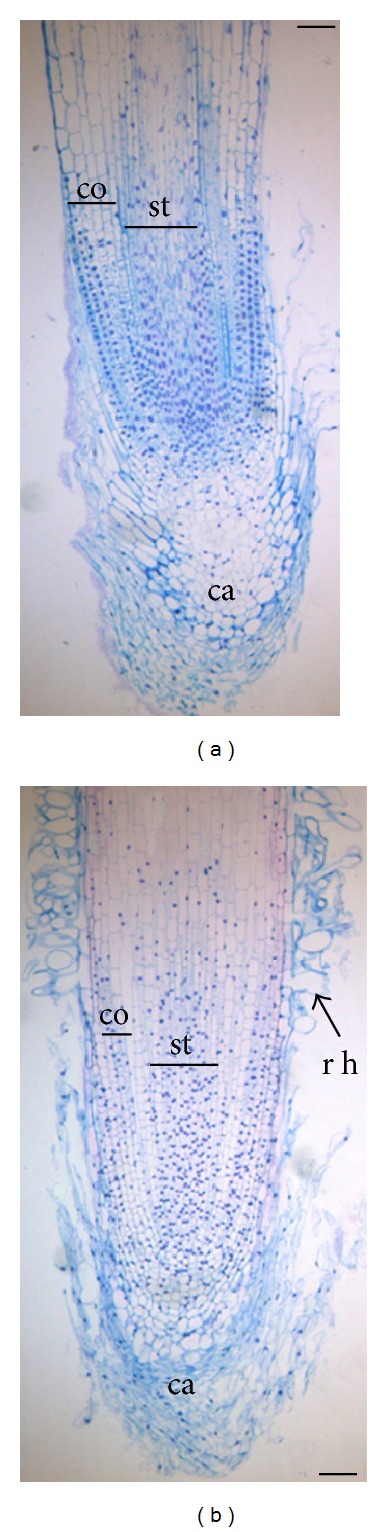
Longitudinal sections of embryonic (a) and adventitious (b) roots of *Olea europaea* L. subsp. *europaea* var. *sylvestris*, stained with periodic acid-Schiff's reagent and 0.5% (w/v) Azur II. The adventitious roots were obtained from *in vitro* rooted cuttings. The embryonic roots come from *in vitro* germinated seeds. ca: calyptra, co: cortex, st: stele, rh: root hairs. (a) = 70 *μ*m; (b) = 80 *μ*m.

To obtain some insights into their pattern formation, developed adventitious roots (Figure 4(b)) were analyzed at histological level and compared with embryonic roots (Figure 4(a)). A typical tissue organization was observed in the root apex of both adventitious and embryonic roots with a meristematic zone covered by a well-developed calyptra, followed in basipetal direction by elongation zone in which stele, cortex, and epidermis were clearly distinguishable (Figures 4(a) and 4(b)). However, it was possible also to notice some interesting differences dealing with the presence, in adventitious roots, of numerous root hairs in the elongation zone of adventitious root compared to embryonic ones. This latter feature clearly highlighted an early differentiation of protoderm cell line in adventitious *versus* embryonic roots.

### 3.4. Features of the *OesARF* and *OesH3* Genes and Deduced Proteins

The cDNA full-length of* OesARF* (*Olea europaea* subsp. *europaeae* var. *sylvestris Auxin Responsive Factor*) showed an open reading frame (ORF) of 525 bp. In* silico *analysis evidenced high homology of *OesARF* with other known homologous genes. The deduced protein, *OesARF*, was of 175 amino acids and blasted in NCBI database (ExPASy Proteomic Tools, http://www.expasy.org/tools/dna.html), it shared the highest identity with that of *Arabidopsis lyrata* (68%), followed by *Arabidopsis thaliana* (63%).

The cDNA full-length of *OesH3* (*Olea europaea* subsp. *europaea* var. *sylvestris* Histone3) showed an open reading frame of 408 bp. In *silico* analysis evidenced high homology of *OesH3* with other known homologous genes. The deduced protein, *OesH3*, was of 136 amino acids and blasted in NCBI database it shared the highest identity with that of *Vitis vinifera* (100%), *Arabidopsis thaliana* (100%), and *Zea Mays* (99%).

### 3.5. *OesARF* and *H3* Are Involved in Adventitious Root Induction

**Figure 5:**
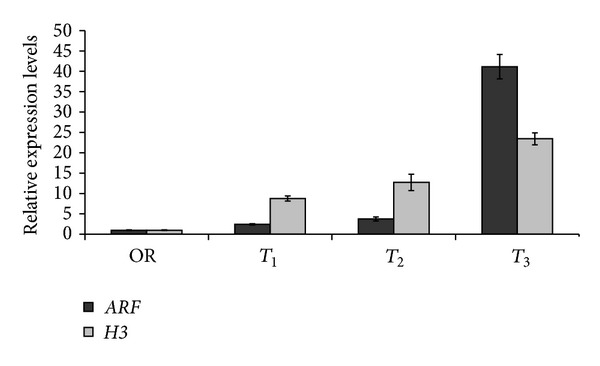
Levels of *OesARF* and *OesH3* erspression in stem cuttings of *Olea europaea* subsp. *europaea* var. *sylvestris* grown for 4 days on different media. Control: stem cuttings were processed immediately after cutting; *T*_1_: OR medium without IBA; *T*_2_: short pretreatment with 14.7 mM IBA sterile solution and inoculation into the OR medium supplemented with 0.5 mg/L IBA; *T*_3_: short pretreatment with 14.7 mM IBA sterile solution and inoculation into the OR medium supplemented with 1 mg/L IBA. The levels of *OesARF* and *OesH3* (log scale) were normalized to H_2_b histone gene and compared with the control condition. Data shown are averages of three biological replicates, with error bars representing SD.

qRT-PCR was used to investigate the expression levels of *OesARF* transcription factor and *OesH3* gene in *oleaster *Sc_s_, grown under sterile conditions for 4 days, on a medium enriched or not with IBA at different concentrations as described in M&M section (Figure 5).

The obtained results indicated that the expression of both *OesARF* and* OesH3* was enhanced in all rooting conditions compared to OR starting point (Figure 5).

In particular, *OesARF* expression slightly increased at *T*_1_ and *T*_2_ conditions and peaked at *T*_3_ condition (30-fold increase). Whereas, *OesH3* transcripts were strongly and differentially enhanced at *T*_1_ (9-fold increase), *T*_2_ (13-fold increase), and *T*_3_ (24-fold increase) conditions.

## 4. Discussion

The aim of this work was addressed to set up an efficient procedure for *in vitro* propagation of *oleaster* which could be very useful for olive culture practice since this wild form is commonly used as root-stock for many cultivars. Moreover, efficient vegetative propagation of *oleaster* could provide a useful tool for a rapid reimplantation of “*Macchia*” vegetation in eroded landscapes.

Previously, an almost satisfactory resumption of vegetative growth was induced in *oleaster* propagated Sc_s_, while a low efficiency was achieved for adventitious rooting which occurred sporadically and required a very prolonged period (i.e., 45 days) [26]. Therefore, the present work was undertaken on one hand to improve vegetative growth performance of Sc_s_ on the other to set up efficient rooting conditions. Concerning vegetative growth, attention was mainly focused on the discovering of cytokinin optimal concentrations. We used two different media widely reported in the literature for *in vitro* propagation of *Olea europaea *species [27, 34, 35]. Using a zeatin concentration of 2.5 mg/L, it was possible to achieve a good balance between an appreciable vegetative growth in both the induction (i.e., about 68% of bud reactivation) and elongation phase (i.e., about 43% axillary shoot elongation) and undesirable callus formation at the cuttings basal which was very limited. Moreover, in line with data in the literature [27, 34, 35], ORZ resulted to be a more suitable medium compared to MSZ.

However, rooting phase constitutes the very critical step of *in vitro* micropropagation process and mainly for woody plants. It is known that auxin plays a major role in root organogenesis [36, 37]. Although auxin IAA (indole-3-acetic acid) was the first plant hormone to be used for stimulating adventitious root formation [38], the “synthetic” auxin IBA (Indole-3-butyric acid) is worldwide used to induce rooting in many plant species and in some cases more efficiently than IAA [13, 39–41]. The greater ability of IBA to promote adventitious root formation compared with IAA has been attributed to its higher stability with respect to IAA in both solution and plant tissue [42].

Taking these data into account, we tested a short IBA pulse, followed by two different IBA concentrations and the rooting efficiency was 70% and 90%, respectively, compared to the rate around 20% or below which characterizes many olive cultivars [23]. Unexpectedly, adventitious rooting was achieved also on medium devoid of IBA and the efficiency was similar to that obtained at the lowest IBA concentration. However, it must be recalled that wounding induces an increase of the Jasmonic acid (JA) level at the cutting end followed by the activation of JA-responsive genes such as cell wall invertase [43, 44]. This in turn might lead to an accumulation of sucrose and after cleavages hexoses at the cutting ends. Therefore adventitious root formation could be related to the presence of these compounds [45, 46].

Noteworthy, at increasing IBA concentrations (i.e., *T*_3_ conditions), rooting efficiency was enhanced as evidenced by the increase of root number × cutting and root length. Analyzing at the histological level the adventitious roots formed at *T*_3_ condition, it was possible to verify that tissue organization and patterning were quite comparable to those of embryonic roots. The only differences observed dealt with a premature protoderm differentiation with abundant root hairs starting from elongation zone. Both these features are consistent with the variation in the root hormonal network induced by *in vitro* cultures [28, 47, 48]. At this respect we may recall that auxin seems to have effects on elongation rather than production of root hairs [49, 50]. However, auxin is also known to increase the production of ethylene in the roots [51]. Notably, ectopic root hairs formation has been detected in ethylene-mutants of *Arabidopsis* [52, 53]. On the other hand, it has been demonstrated that in *Arabidopsis* seedlings growing in presence of an inhibitor of ethylene synthesis, the addition of IAA allowed the normal formation of root hairs likely by inducing ethylene production [54]. Therefore, the abundance of hairs along the adventitious roots of *T*_3_ cuttings grown in the presence of auxin may include these mechanisms. The correct developmental pattern of adventitious root system of *T*_3_ cuttings was further supported by their successfully overcoming acclimation phase in pots.

Finally we also report that during rooting induction phase the expression level of two genes (i.e., *OesARF* and* OesH3*), involved in auxin signaling and cell cycle progression respectively [15, 21, 25], increased and such increase was linearly related to rooting efficiency. Both these interplaying events are essential for the resumption of cell proliferation during induction phase. Therefore, this result, besides being consistent with the capacity of auxin to modulate transcript levels of numerous genes [14] demonstrated that a threshold in the expression level of selected genes resulted to be essential for assuring a good performance of rooting process.

In conclusion, in the present work we demonstrated for the first time that the propagation of *oleaster* by seedling-derived cuttings is feasible with highly satisfactory results. We also suggest that the use of selected genes *OesARF* and *OesH3* as markers of the induction phase could be very useful for setting up efficient culture conditions along the rooting process, thus increasing micropropagation efficiency.

## Figures and Tables

**Table 1:** Effects of different medium on the vegetative growth of seedling-derived cuttings (Sc_s_) of *Olea europaea* L. subsp.* europaea* var*. sylvestris* after 45 days of *in vitro* culture. MSZ_2.5_ MSZ_5 _= Murashige and Shoog medium + 2.5 mg/L (11.5 µM) and 5 mg/L (23 µM) *trans*-zeatin, respectively; ORZ_2.5_ ORZ_2.5_; olive medium + 2.5 mg/L (11.5 µM) and 5 mg/L (23 µM) *trans*-zeatin, respectively. Means with common letters are not significantly different at *P* ≤ 0.05, according to Turkey's Honestly Significant Difference (HSD) test.

Medium	Sample number	Budding (%) (no. of cuttings with open buds/total no. of cuttings × 100)		Shooting (%) (no. of cuttings with lateral shoots/total no. of cuttings × 100)		Shoot length (mm)	
MSZ_2.5_	50	67.7 ± 1.0	a	37.5 ± 0.8	a	9.2 ± 0.8	a
MSZ_5_	50	54.0 ± 1.2	b	32.3 ± 1.2	b	9.0 ± 0.7	a
ORZ_2.5_	50	69.0 ± 2.5	a	48.0 ± 1.2	c	10.5 ± 1.0	a
ORZ_5_	50	56.3 ± 1.2	b	40.5 ± 1.3	d	9.2 ± 0.9	a
